# Supplementary material for: Dynalign II: common secondary structure prediction for RNA homologs with domain insertions
Source: Nucleic Acids Res. 2014 Nov 21;42(22):13939–48. doi: 10.1093/nar/gku1172 (PMC4267632; doi:10.1093/nar/gku1172)
Supplement: SUPPLEMENTARY DATA [file supp_gku1172_nar-02021-z-2014-File012.zip › manual/Text/index.html]

RNAstructure Command Line Help


|  |  |  |
| --- | --- | --- |
|  | RNAstructure Command Line Help  Version 5.7 | - Contents - Index |
| RNAstructure is a product of the Mathews Lab, University of Rochester Medical Center, Department of Biochemistry and Biophysics.  Continued development of RNAstructure is made possible by the support of NIH grant R01GM076485.  General Help   - Instructions for building the text interfaces - General instructions for specifying thermodynamic parameters - I/O file formats   Specific Interface Help  AllSub  Generate all suboptimal nucleic acid structures within a small free energy increment of the lowest free energy structure.  bifold and bifold-smp  Bimolecular folding of nucleic acids with intramolecular pairs allowed.  bipartition and bipartition-smp  Bimolecular partition function calculation of nucleic acids without intramolecular pairs allowed.  CircleCompare  Compare two structures for the same sequence, with the nucleic acid backbone arranged around a circle to facilitate easy comparisons.  ct2dot  Generate a dot bracket file from a CT file. The CT file can be regenerated from the dot bracket file using dot2ct (below).  dot2ct  Generate a CT file from a dot bracket file. The dot bracket file can be regenerated from the CT file using ct2dot (above).  draw  Draw a structure (or structures) in Postscript from a CT file. This can be done a variety of ways: annotated, unannotated, circularized, or a combination of these.  DuplexFold  Bimolecular folding of nucleic acids without intramolecular pairs allowed.  dynalign\_ii, dynalign, dynalign\_ii-smp, and dynalign-smp  Find a secondary structure common to two sequences.  DynalignDotPlot  Generate a free energy dot plot from a Dynalign calculation, exported as a Postscript file.  efn2 (Energy Function 2) and efn2-smp  Determine the folding free energy change of a structure.  EnergyPlot  Generate a free energy dot plot from a folding calculation save file, exported as a Postscript file.  EnsembleEnergy  Calculate the ensemble energy of structures from base pair probabilities or sequence data.  Fold and Fold-smp  Predict the lowest free energy structure for a nucleic acid sequence, as well as a set of low free energy structures.  MaxExpect  Predict the maximum expected accuracy structure for a nucleic acid sequence.  multifind  Find non-coding RNAs in sequence alignments.  multilign  Find secondary structures common to multiple sequences (more than two).  NAPSS  Use NMR data to improve the prediction of an RNA secondary structure.  oligoscreen and oligoscreen-smp  Determine folding thermodynamics for a set of oligonucleotides.  OligoWalk  Calculate thermodynamic features of sense-antisense hybridization and predict free energy changes of oligonucleotides binding to target RNA.  partition and partition-smp  Predict nucleic acid base pairing probabilities using a partition function.  partition-cuda  Predict nucleic acid base pairing probabilities using a partition function in parallel using a GPU.  PARTS  Find a secondary structure common to two nucleic acid sequences, a sample of those structures, and/or the probability of pairing for common base pairs.  ProbabilityPlot  Generate a base pairing probabilities dot plot from a partition function save file, exported as a Postscript file.  ProbablePair  Predict structures from base pair probabilities, using a threshold to include only pairs within a specific range of probabilities.  ProbKnot  Predict a maximum expected accuracy structure, including pseudoknots.  refold  Use a save file to predict a new set of suboptimal structures.  RemovePseudoknots  Remove pseudoknots from an RNA structure, outputting the most likely pseudoknot-free structure.  scorer  Calculate sensitivity and positive predictive value for two structures being compared.  ShapeKnots  Predict a secondary structure that can contains pseudoknots, restrained by SHAPE mapping data.  stochastic and stochastic-smp  Generate a representative ensemble of structures.  TurboFold  Fold multiple sequences into their optimal conformations, using one of three different folding modes. | | |
| Visit The Mathews Lab RNAstructure Page for updates and latest information. | | |
